# Mental health outcomes of hospital staff during the COVID-19 pandemic in Iran

**DOI:** 10.1186/s12913-023-10430-w

**Published:** 2023-12-20

**Authors:** Sahar Salehi, Maryam Jamali, Mahdi Shafiei Neyestanak, Milad Safaei Amjaz, Vali Baigi, Mir Saeed Yekaninejad

**Affiliations:** 1 Department of Biostatistics and Epidemiology, Faculty of Public Health, Kerman University of Medical Sciences, Kerman, Iran; 2 Imam Hossein Hospital, Isfahan University of Medical Sciences, Golpayegan, Iran; 3 Department of Epidemiology and Biostatistics, School of Public Health, Tehran University of Medical Sciences, Tehran, Iran; 4 Faculty of Razi Nursing and Midwifery, Kerman University of Medical Sciences, Kerman, Iran; 5 Sina Trauma and Surgery Research Centre, Tehran University of Medical Sciences, Tehran, Iran; 6 Department of Integrative Structural and Computational Biology, Scripps Research, San Diego, USA

**Keywords:** Mental health, COVID-19, Outbreak, Health care provider, Clustering, K-Means

## Abstract

**Background:**

The COVID-19 pandemic, which had recorded 769 million cases and resulted in 6.95 million deaths by August 2023, has put pressure on healthcare systems. Frontline medical professionals face stress, potentially leading to health challenges. This research aimed to examine the mental health of staff during the COVID-19 pandemic.

**Methods:**

This cross-sectional descriptive-analytical study was conducted in several hospitals in Tehran, Kerman, and Golpayegan between 2021 and 2022. The study encompassed a population of 1,231 nurses and physicians. Data collection was done using the General Health Questionnaire-28 (GHQ-28). We applied the K-means clustering algorithm to unveil hidden patterns within the data and extract valuable insights from participants' responses to the GHQ-28. This method was chosen because our dataset lacked explicit labels, making grouping individuals with similar characteristics necessary. The primary aim was to delineate how the COVID-19 pandemic affected the mental health of hospital staff and identify which factors played a more significant role in this process.

**Results:**

We have observed that Cluster two exhibits the highest scores in response to the GHQ-28 questions, indicating a more significant degree of mental distress. Within this cluster, 83.0% of individuals identify as female, 71.0% hold bachelor's degrees and 42.8% are nurses who have experienced the most substantial impact. Among these individuals, 90.4% did not have a history of smoking. Additionally, 59.7% are married, suggesting that these mental health issues may also affect their families.

**Conclusion:**

Given that the most critical subscale is related to anxiety/insomnia within the second cluster, it is necessary to implement management plans aimed at appropriately redistributing night shifts to improve employee health.

## Background

COVID-19 is a disease that has caused numerous deaths and cases since December 2019. The most recent global data, as of August 2023, indicate that this disease has affected approximately 769 million people and has resulted in over 6,955,000 fatalities worldwide [1]. According to Iran's Ministry of Health and Medical Education, there have been 7,613,000 confirmed cases of this disease in Iran, with the number of deaths surpassing 146,000 as of August 2023 [2]. It's important to note that the world's population exceeds 8 billion, while Iran's population is over 85 million [3, 4].

The COVID-19 pandemic has significantly impacted countries’ economic, political, cultural, and social aspects worldwide. This pandemic has led to an unprecedented demand for healthcare services worldwide, resulting in medical staff managing a heavier workload under stressful conditions [5].

Medical staff are consistently exposed to stress and are at risk of experiencing mental health problems due to the nature of their profession. They must treat patients with infectious and life-threatening diseases or suspected conditions, manage an overwhelming workload, deal with a shortage of personal protective equipment, face media scrutiny, and cope with a lack of social support [6–8]. In 2021, Zakari et al. conducted a study to investigate mental health outcomes in healthcare workers (HCWs) in Rafsanjan, Iran, using the General Health Questionnaire (GHQ-28) and concluded that 45.5% of the participants exhibited psychological disorders. Therefore, it is recommended to enhance the psychological skills of HCWs through counseling and training programs [9].

Long-term exposure to stressful situations can weaken the immune system, reduce the body's ability to fight diseases, and lead to complications such as anger, insomnia, fear of disease (even for non-high-risk individuals), social isolation, alcohol abuse, smoking, and post-traumatic stress disorder (PTSD) [6, 10]. These complications can ultimately diminish the quality of healthcare services, increase the frequency of medical errors, reduce patient safety, and jeopardize health organizations' overall performance and effectiveness [11–13].

To manage the mental health status of hospital staff, it is advisable to categorize them to identify those at the highest risk. Utilizing modern tools and emerging scientific fields can provide quick answers. Therefore, this study employs advanced data analysis methods from data science to analyze the collected data and solve the research questions.

Clustering is one of the most reliable methods for discovering similar features in a dataset. In this method, data with similar characteristics are grouped into clusters that are distinct from other data. Clustering is an exploratory process to identify meaningful structures or patterns within a dataset. It is an unsupervised learning method, meaning that the algorithms used in this method can detect patterns in untagged data by identifying similar behaviors.

Alam et al. used K-means clustering to categorize university students based on their psychological health scores to evaluate the mental health status of Bangladeshi university students during the lockdown period [14].

In this study, we employed the K-means clustering algorithm, a method that relies on the data's inherent structure, consisting of responses to the GHQ-28 questionnaire, without the need for predefined thresholds. The primary objective of this approach is to assign individuals to groups in a manner that meets specific criteria. Typically, clustering criteria aim to minimize differences within each group or maximize the distinctions between different groups equivalently.

This study aimed to compare the mental health status of medical staff during the COVID-19 pandemic in several cities in Iran. The main advantage of this study over other similar research lies in using data clustering methods. Consequently, the study findings can assist health policymakers and decision-makers understand the current situation and take appropriate measures to support medical staff and enhance their mental health.

## Method

### Patients and setting

This cross-sectional study was conducted in 11 Tehran, Kerman, and Golpayegan hospitals in 2021–2022. In terms of geographical location, these three cities represented the cities of Iran. The study population consisted of all nurses and physicians working in Afzalipour, Payambar-e Azam, Shafa, and Bahonar hospitals of Kerman, Imam Hossein Hospital of Golpayegan, and Sina, Razi, Shariati, Farabi, Bahrami, and Milad hospitals of Tehran and we sent a questionnaire (between October 2021 and January 2022) to 1968 people.

The only inclusion criterion was that all nurses and physicians with informed consent and willingness to fill out the research questionnaire. The exclusion criterion was a history of psychiatric diseases and taking psychiatric medicines. To identify this category of people, in the initial questions of the questionnaire, people were asked about their history of psychiatric diseases and taking psychiatric medication. The questionnaire was completed automatically for the samples who answered "yes" to the previous mental health problems question, and the questions related to the level of mental health were not shown to them.

### Data collection

The online version of the General Health Questionnaire-28 (GHQ-28) was used for data collection. Taqavi’s article assessed the validity and reliability of the GHQ-28 questionnaire [15]. The contact information of all doctors and nurses eligible to participate in the study was obtained from the relevant units in the studied hospitals.

The GHQ-28 questionnaire is a 28-question survey used to identify mental disorders in the general population and is divided into four subscales: somatic symptoms, anxiety/insomnia, social dysfunction, and severe depression. The total score ranges from 0 to 84 (on a Likert scale), with higher scores indicating higher levels of distress [16]. A score between 0 and 22 was considered “none or the lowest level of mental disorders.” A score between 23 and 40 was considered a “low level of mental disorders.” A score between 41 and 60 was considered an “average level of mental disorders,” and a score between 61 and 84 was considered a “severe level of mental disorders.”

All physicians and nurses who met the inclusion criteria were contacted from the studied hospitals. Then, the questionnaire link was sent to them via social networks (Telegram, WhatsApp, and Email) and SMS. Sampling was done using the snowball method. After reminding each participant about completing the questionnaire twice at a one-week interval, those who did not respond were excluded from the study, indicating their unwillingness to participate. The questionnaire was designed so each participant could fill it out only once.

### Statistical method

The variables used in this study include gender, marital status, smoking, direct exposure to COVID-19 patients, chronic diseases, and specific jobs in the hospital. Then, data science methods, especially data clustering, were used to classify data based on the research objectives. To achieve this, clustering algorithms were employed to identify appropriate data patterns. As a result, data representing specific features were grouped into a single cluster. The number of clusters was determined based on data extracted from questionnaires and expert opinion. The clustering algorithms also provided tags for each cluster. In addition, the reason for assigning each person to any of the clusters was determined by drawing a diagram. All the algorithms mentioned above were performed in R-Studio.

In this study, we used the K-Means clustering algorithm. The k-means algorithm is generally the most well-known and widely used clustering method. Although it is an unsupervised learning method in pattern recognition and machine learning, the k-means algorithm and its extensions are always affected by initializations with a necessary number of clusters a priori.

K-means makes inferences from datasets using merely input vectors without referring to known or labeled outcomes. Every data point is allocated to a cluster by minimizing the in-cluster sum of squares. In other words, the K-means algorithm identifies the k number of centroids and then allocates every data point to the nearest cluster while keeping the centroids as small as possible. The ‘means’ in K-means refers to averaging of the data; that is, finding the centroid.

After implementing k-means clustering, we assigned a cluster ID to each sample to find the similar characteristics of samples within each cluster. Before presenting the results, an insightful bar plot showing the relation between the average score of responses to GHQ-28 and cluster members is shown in Fig. 2.

### Clustering

Grouping objects is crucial in various fields. For instance, patients with shared symptoms are categorized by diseases. Classification is divided into supervised (labeled) and unsupervised (unlabeled) methods. Supervised builds classifiers to predict labels, while unsupervised clustering finds natural groupings without labels. Learning is vital in both processes [17]. Rokach explains that clustering involves grouping data patterns into subsets with similar patterns. This arrangement creates a structured assessment, effectively characterizing the sampled population [18].

When we have a large collection of data points, each representing an object, person, or event, clustering algorithms aim to partition these data automatically points into groups, or “clusters,” where items within the same cluster are more similar to each other compared to those in different clusters. This similarity is usually measured using mathematical distance metrics that quantify how close or far apart data points are in the feature space.

K-Means [19, 20] clustering is a widely used unsupervised machine learning algorithm designed to partition a dataset into distinct groups or clusters based on similarity. It’s a straightforward yet powerful technique effective when the number of clusters is known in advance. Some advantages of K-means are Simplicity and efficiency, as well as Scalability to large datasets and Interpretability of results.

K-means has some key steps that we want to address here.

- Initialization: The algorithm randomly selects K data points as cluster centroids. These centroids serve as the initial centers of the clusters.
- Assignment: Each data point in the dataset is assigned to the nearest cluster centroid. This assignment is based on a distance metric, commonly the Euclidean distance, which measures the distance between data points and centroids.
- Updating Centroids: After assigning all data points to clusters, the algorithm recalculates the centroids of these clusters. The centroid of a cluster is the mean of all data points belonging to that cluster.
- Re-Assignment and Updating: Steps two and three are repeated iteratively. Data points are reassigned to the nearest centroids, and centroids are recalculated based on the new assignments. This process continues until the centroids no longer change significantly or a maximum number of iterations is reached.
- Convergence: Eventually, the algorithm converges to a point where data points are stably assigned to clusters and centroids remain relatively constant.

Clustering can offer distinct advantages over traditional methods when studying medical staff's mental health during the pandemic. This can give us Unbiased Grouping.

Traditional methods might require predetermined criteria or labels for classifying members into clusters, which can be limiting and biased. Clustering, on the other hand, can group individuals based on shared patterns without requiring predefined categories.

Also, with the help of clustering, we can find complex patterns and variations in mental health states that might not fit predefined classifications.

Interestingly, with the help of unsupervised learning, we can identify unknown factors. Clustering can reveal novel patterns and correlations that traditional methods might miss.

## Results

The total sample size used was 1,231 people, 74.5% (*n* = 918) female. The majority (66.4%) held a bachelor's degree (*n* = 818), 15.4% had a master's degree, and 5.4% possessed a Ph.D. or higher. The percentage of married individuals in the sample was 60.0, or *n* = 750. Additionally, 91.4% of the people in the research had no smoking history. Among all hospital staff who contributed to this research, 73.5% (*n* = 905) directly interacted with patients with COVID-19. Also, 1,113 (90.4%) did not have chronic diseases such as diabetes, obesity, or high blood pressure. The distribution of their specific jobs in the hospital is shown in Table 1.

**Table 1:** baseline characteristics of participants

Characteristics	N (%)
**Gender**	
Male	313 (25.4)
Female	918 (74.6)
**Marital Status**	
Single	481 (39.1)
Married	750 (60.9)
**Education**	
Bachelor	818 (66.4)
Masters	190 (15.4)
Ph.D. and higher	67 (5.4)
Diploma	131 (10.6)
High school	25 (2.0)
**Smoking**	
No	1125 (91.4)
Yes	106 (8.6)
**Direct Exposure to COVID-19 Patient**	
No	326 (26.5)
Yes	905 (73.5)
**Chronic Diseases**	
No	1113 (90.4)
Yes	118 (9.6)
**Job**	
Physician	131 (10.6)
Nurse	507 (41.1)
Laboratory Expert	34 (2.7)
Midwife	110 (8.9)
Nurse Assistance	44 (3.5)
Other	405 (32.9)
**Mean total score**	26

**Fig. 1:**
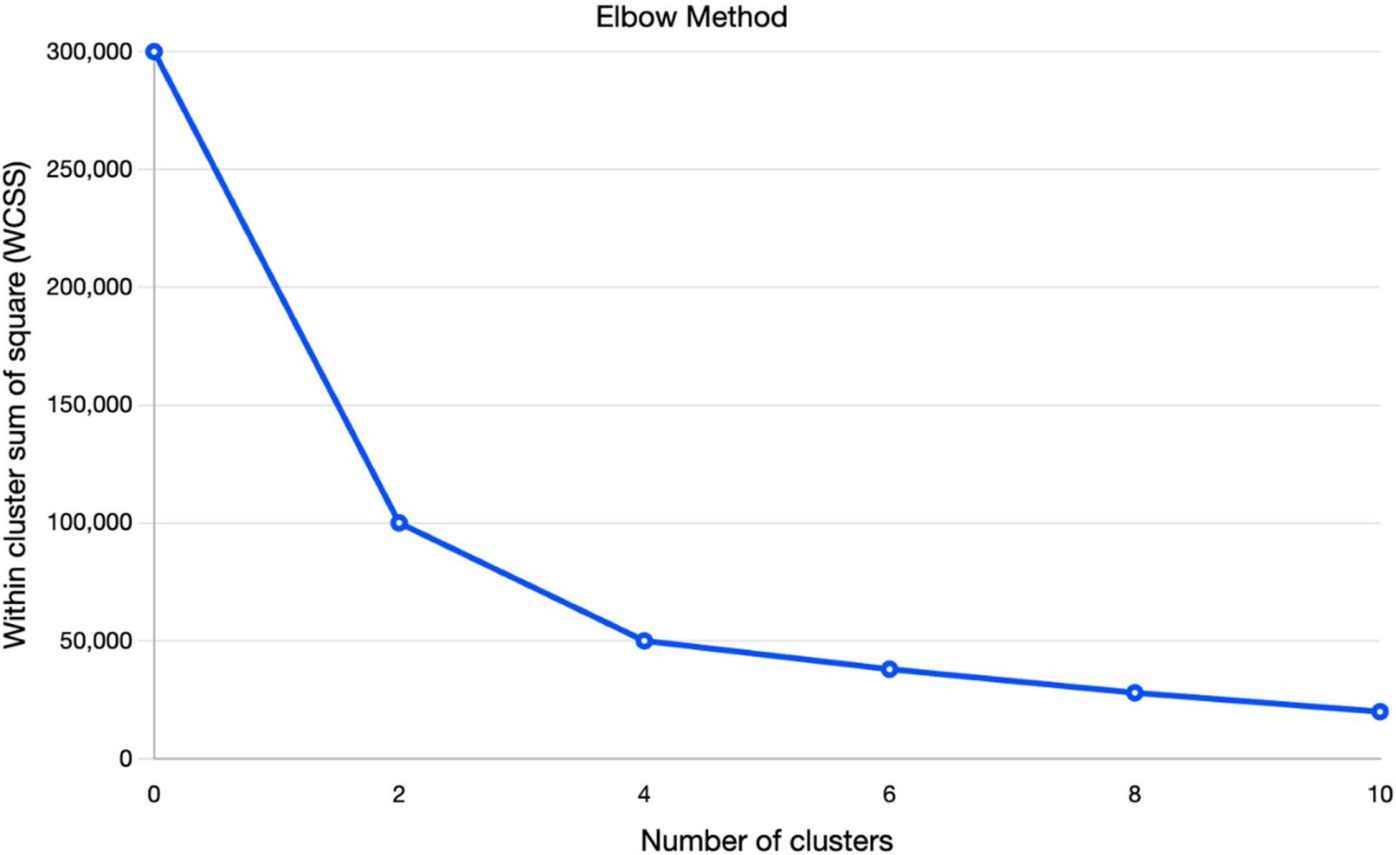
The elbow method plot to find out the proper number of clusters

**Fig. 2:**
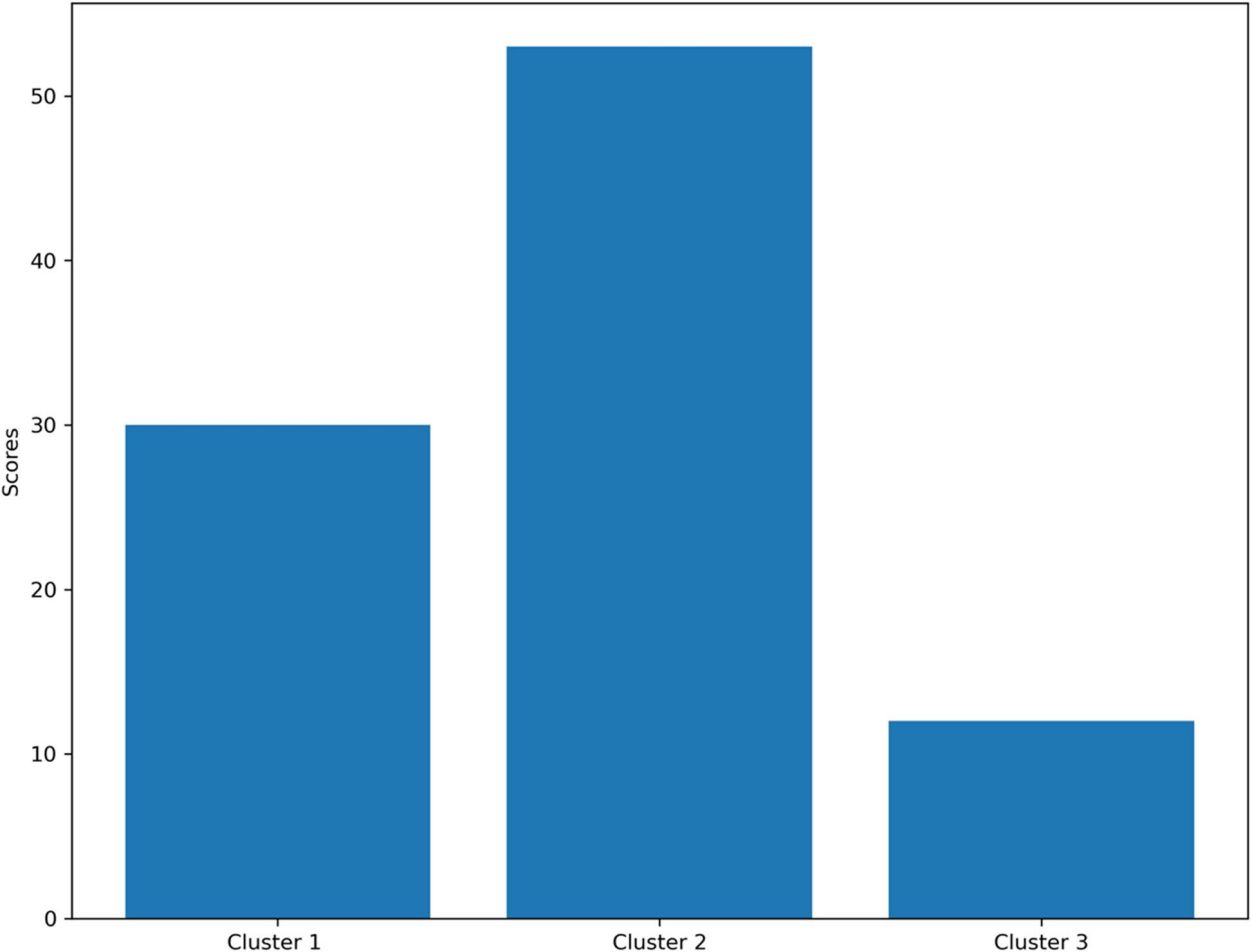
Mean total score for each individual based on their responses to the GHQ-28 questionnaire

The average score of GHQ-28 responses was 26. Before implementing k-means clustering, we utilized the Elbow method. This curve is depicted in Fig. 1, which suggests that it's more suitable to use K = 3 clusters based on our dataset. In Fig. 2, we also present the average scores obtained from responses to the GHQ-28 questionnaire.

The model’s output reveals that our first cluster comprises 506 individuals, while Cluster 2 consists of 189 people, and Cluster 3 encompasses 536 hospital staff members. These clusters were formed based on their members' shared characteristics and behavior. Detailed features of these three clusters are presented in Table 2.

**Table 2:** Clustering according to baseline characteristics, N (%)

C**haracteristics**	**Cluster 1**	**Cluster 2**	**Cluster 3**	***P*-value**
**Gender**				
Male	107 (21.1)	32 (16.9)	174 (32.4)	< 0.001
Female	399 (78.8)	157 (83.0)	362 (67.5)	
**Marital Status**				
Single	200 (39.5)	76 (40.2)	205 (38.2)	0.86
Married	306 (60.4)	113 (59.7)	331 (61.7)	
**Education**				
Bachelor	317 (63.2)	135 (71.0)	358 (66.2)	0.04
Masters	89 (17.7)	30 (15.7)	80 (14.8)	
Ph.D. and higher	38 (7.5)	7 (3.6)	21 (3.8)	
Diploma	48 (9.5)	14 (7.3)	69 (12.2)	
High school	9 (1.7)	4 (2.1)	12 (2.2)	
**Smoking**				
No	459 (90.7)	171 (90.4)	494 (92.1)	0.64
Yes	47 (9.2)	18 (9.5)	42 (7.8)	
**Direct Exposure to COVID-19 Patient**				
No	108 (21.3)	40 (21.1)	178 (33.2)	< 0.001
Yes	398 (78.6)	149 (78.8)	358 (66.7)	
**Chronic Diseases**				
No	450 (88.9)	167 (88.3)	495 (92.3)	0.11
Yes	56 (11.0)	22 (11.6)	41 (7.6)	
**Job**				
Physician	67 (13.2)	16 (8.4)	38 (7.0)	< 0.001
Nurse	195 (38.5)	81 (42.8)	226 (42.1)	
Laboratory Expert	23 (4.5)	6 (3.1)	9 (1.6)	
Midwife	46 (9.0)	25 (13.2)	39 (7.2)	
Nurse Assistance	14 (2.7)	2 (1.0)	28 (5.2)	
Other	161 (31.8)	59 (31.2)	196 (36.5)	
**Mean total score**	30	52	13	< 0.001

Based on the above results, Cluster 2 exhibits the highest scores on the GHQ-28 questionnaire, with an average score of 52, indicating a higher level of mental distress. Within this cluster, 83% are female, 71% hold bachelor's degrees and 42.8% are nurses who appear to have experienced the most significant mental distress. and 90.4% had no history of smoking. Additionally, 59.7% are married, suggesting potential ripple effects on their families.

To identify which variables significantly differ among the clusters, we conducted a chi-square test with a significance level of 5%. The results indicate that gender, education, direct exposure to COVID-19 patients, and occupation are significant at *p* < 0.05. These findings are presented in Table 3.

**Table 3:** GHQ domain descriptive table

Cluster-ID		Somatic symptoms	Anxiety/insomnia	Social dysfunction	Severe depression	N
**1**	Mean	8.37	8.51	8.40	4.41	506
	SD^a^	2.95	2.88	2.28	3.42	
**2**	Mean	12.70	14.58	12.75	11.45	188
	SD	3.79	3.38	3.23	4.99	
**3**	Mean	3.71	3.20	5.60	0.91	536
	SD	2.21	2.25	2.22	1.42	
**Total**	Mean	7.01	7.13	7.85	3.96	1229
	SD	4.28	4.83	3.44	4.71	

^a^Std. Deviation

To compare mean scores across clusters and GHQ domains through statistical analysis, we initially identified the most suitable test for inter-cluster comparisons. Following this, we assessed the normality of the datasets within each cluster to understand their distribution.

To address this, we employed a Normal Q-Q plot, a graphical tool used for assessing the normality of a dataset, and we also compared the boxplots. Its primary purpose is to determine whether the data distribution closely conforms to a standard normal (Gaussian) distribution or deviates from it.

Based on the Q-Q plots, we concluded that the distributions within the clusters do not adhere to normal, as indicated by significant deviations in the tails. This substantial evidence strongly suggests a departure from the normal distribution assumption.

Accordingly, we have employed a robust non-parametric statistical test, namely the Kruskal–Wallis test, to compare the three clusters effectively.

After conducting the Kruskal–Wallis test, The *p*-value was less than 0.001, indicating statistical significance, and there is a difference among the medians of the clusters being compared.

**Fig. 3:**
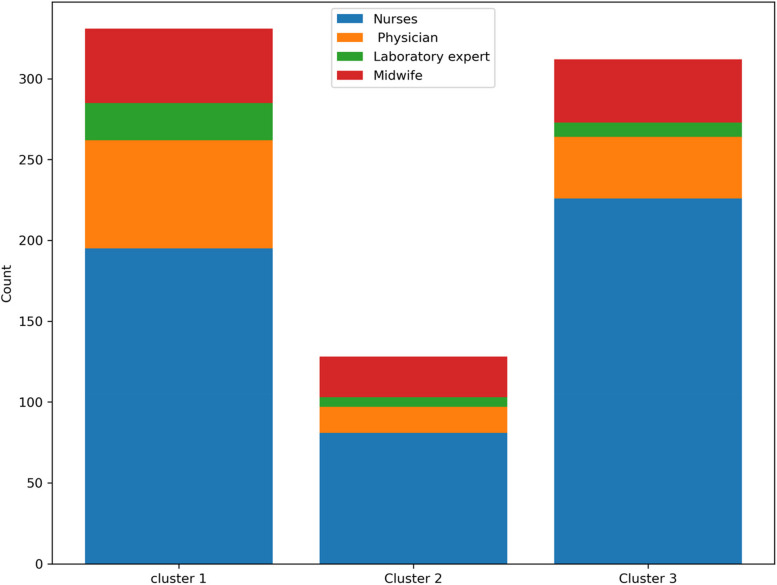
Distribution of jobs in each cluster

To enhance clarity, we’ve included some helpful charts below. These visuals give a full view of the clusters and make it simpler to understand the demographic trends. In Fig. 3, we can see the job distribution in each cluster. From this chart, it's clear that there are more nurses than any other group.

**Fig. 4:**
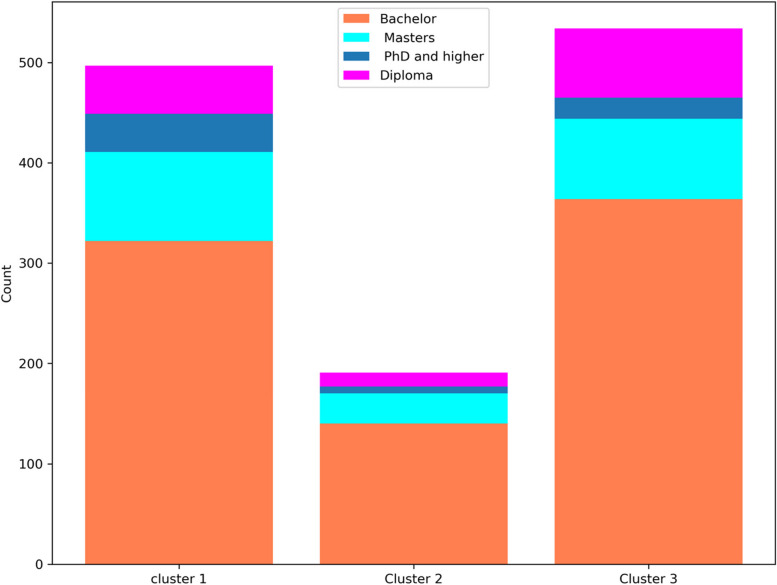
Distribution of education in each cluster

In Fig. 4, the graphic representation illustrates the distribution of education levels within each cluster. Observing this plot, it becomes evident that a significant proportion of individuals within the clusters possess a bachelor's degree, indicating the prevalence of this educational attainment among cluster members.

**Fig. 5:**
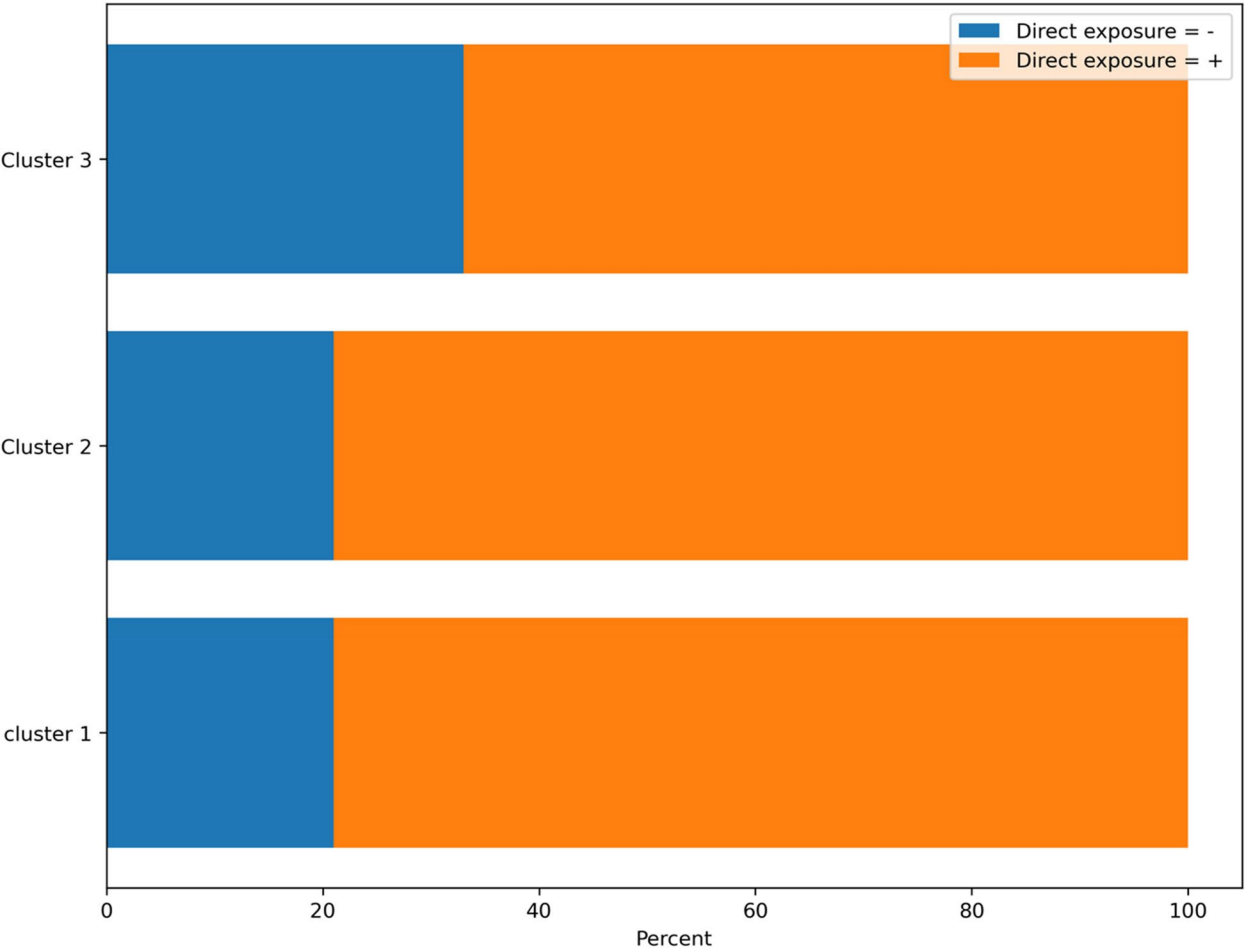
Direct exposure status of hospital staff with Covid-19 patients

In Fig. 5, we sought to graphically show what percentage of our samples were directly exposed to patients with COVID-19.

## Discussion

Due to the unprecedented demand for healthcare services during the COVID-19 pandemic and the absence of effective treatment, healthcare workers faced increasingly heavy workloads and stressful situations [5, 21]. Therefore, this study aimed to analyze the health status of hospital staff during the COVID-19 pandemic in Iran. This study provides a unique perspective on the experiences and impact of the COVID-19 pandemic on the mental health of physicians and nurses using a novel analysis method called clustering, which has not been used in Iran previously.

In general, when considering the mean GHQ score of all samples (mean = 26), it appears that employees of health centers had a low level of mental disorders despite 73.5% of the samples dealing with COVID-19 patients. After implementing data clustering, three clusters emerged. According to the results, the mean GHQ score of the first cluster indicated a low level of mental disorders (mean = 30), with 78.6% of samples (398 individuals) in this cluster directly involved with COVID-19 patients. In this cluster, the subscales of somatic symptoms, anxiety/insomnia, and social dysfunction were worse than severe depression.

Furthermore, out of the total of 189 individuals in the second cluster, 149 people (79%) had a history of direct exposure to a confirmed positive COVID-19 patient. The most critical issue in this cluster was the anxiety/insomnia subscale. Finally, the mean GHQ score of the third cluster indicated the lowest level of mental disorders (mean = 13), with 66.7% of samples (358 individuals) in this cluster dealing directly with COVID-19 patients. In this cluster, the subscales of somatic symptoms, anxiety/insomnia, and severe depression were in a better position than social dysfunction.

Since the clusters were formed based on the similar characteristics and behaviors of their members, more samples were involved in dealing with COVID-19 patients in the clusters with low (mean = 30) and average levels (mean = 52) of mental disorders compared to the cluster with the lowest level of disorders (mean = 13). This involvement could exacerbate the mental disorders of health staff in the first and second clusters. However, the lowest level of such disorders (mean = 13) was observed in a higher percentage of samples (66.7% or 358 individuals) dealing with COVID-19 patients in the third cluster. In other words, the samples of the third cluster had different responses to COVID-19 patients compared to the samples of the other two clusters. Given that the most critical subscale was related to anxiety/insomnia in the second cluster, management plans are needed to distribute night shifts to improve employee health properly.

The study examined the vulnerability of nurses, particularly females, based on factors such as education, direct exposure to COVID-19 patients, occupation, and marital status. The researchers compared mental health levels between nurses in COVID-19 hospitals and other hospitals. The results showed significantly lower mental health levels among nurses in COVID-19 hospitals, suggesting a higher prevalence of mental disorders among them. This difference may be attributed to the evolving nature of the pandemic, with varying levels of mental disorders at different stages, including the initial outbreak and the vaccination period.

The study identified direct exposure to COVID-19 patients as a significant variable affecting mental health, which differed from a previous study [22]. A considerable proportion of participants in specific clusters had direct contact with COVID-19 patients, potentially increasing their risk of mental disorders. The variation in results between the current study and the previous one could be attributed to differences in time, the evolving understanding of the disease since its onset in April 2020, and the vaccination of medical staff during the present study. Moreover, the present study had a larger sample size, including 1231 individuals from various positions in health centers, while the previous study included only 122 nurses.

Another significant observation from the clustering results pertains to the influence of direct exposure to COVID-19 patients on the average total score obtained by each individual in response to the GHQ-28 questionnaire. For instance, in clusters one and two, 79 percent reported direct exposure to patients, the corresponding mean total scores were 30 and 52, respectively. These scores notably exceeded the mean total score of cluster number three, where 66.7 percent reported direct exposure and yielded a score of 13. This interpretation suggests a clear association between direct patient exposure and a higher incidence of mental disorders in hospital staff.

Davarinia Motlagh Ghouchan et al. compared the mental health of nurses in COVID-19 hospitals with those in other hospitals. According to their results, nurses in COVID-19 hospitals had significantly lower levels of mental health, while no significant differences were found between these nurses and those in other hospitals. The variance between the findings of this study and those of the study by Davarinia Motlagh Ghouchan et al. lies in the intensity of mental disorders experienced by hospital staff. They encountered different levels of mental disorders at different times due to the unknown nature of this disease at its onset in April 2020 and the beginning of vaccination during this study. Davarinia Motlagh Ghouchan et al. also sampled 122 nurses, whereas this study included 1231 individuals from various health center positions [22].

Tella et al. [23] studied the mental health of healthcare workers during the COVID-19 pandemic in Italy and concluded that employees in COVID-19 wards experienced higher levels of depression and PTSS symptoms. According to the regression analysis results, they reported that working in COVID-19 wards and being female were among the vulnerability factors for depression symptoms. However, women with spousal support (i.e., gender and marital status) were identified as protective factors for maintaining better mental health. The difference in research timelines could also explain the disparities between the results reported by Tella et al. and the findings of this study.

Additionally, Spoorthy et al. in their study concluded that several demographic variables, such as age, gender, workplace, work sector, profession, and psychological variables like self-efficacy and poor social support, were associated with increased stress, depressive symptoms, anxiety, and insomnia in healthcare workers [24]. There is increasing evidence suggesting that COVID-19 can be an independent risk factor for stress in healthcare workers. However, after universal vaccination and better control of conditions, Fattori et al. showed in their study that two years after the start of the coronavirus epidemic, the mental health of healthcare workers gradually improved over time [25].

Based on the findings of our study, gender (specifically being female), education level, direct exposure to COVID-19 patients, and occupation were identified as variables that exhibited significant differences within the clusters. These results are consistent with the aforementioned study, where being female and direct exposure to COVID-19 patients were recognized as vulnerability factors.

Understanding the mental health status of hospital employees is of utmost importance. Low mental health not only contributes to personal problems but also has repercussions on families, society, service quality, the occurrence of medical errors, patient safety, and client satisfaction. Given the global impact of COVID-19, it is crucial to be attentive to potential mental health issues and leverage pandemic-related experiences for the future. This study uses data clustering algorithms to explore the relationship between the mental health of healthcare personnel and the COVID-19 pandemic. This research provides essential evidence for promoting mental health among healthcare workers and offers a clear understanding of both risk and protective factors that play a role in reducing the prevalence of mental disorders. These insights are valuable for policymakers and healthcare decision-makers, enabling them to take appropriate measures to support healthcare staff, their families, and patients.

This study's strengths include a substantial sample size, encompassing 1,231 doctors and nurses, as well as the utilization of data clustering techniques. Nonetheless, a few limitations were encountered, including Interpreting relationships due to the nature of cross-sectional study should be done with caution. Another limitation of the present study was that due to the limited time of doctors and nurses, it was possible for their response rate to be low, so we tried to reduce this limitation by using an online questionnaire and increase the response rate. Additionally, there was some data loss due to technical issues with the questionnaires for a brief period.

## Conclusion

Low levels of mental health among healthcare center workers can lead to a decrease in the quality of services. Analyzing the mental health subscales in both groups can help identify areas where hospital personnel require training and empowerment, particularly during critical situations like a pandemic. Consequently, improvement strategies can be devised to ultimately enhance the quality of services and the overall health of hospital staff.

Notably, the most critical subscale pertains to anxiety and insomnia within the second cluster. To address this issue and improve employee well-being, management should consider implementing measures such as a more balanced distribution of night shifts.

## Data Availability

The datasets analyzed during the current study are available from the corresponding author upon reasonable request.
